# Nanopolystyrene translocation and fetal deposition after acute lung exposure during late-stage pregnancy

**DOI:** 10.1186/s12989-020-00385-9

**Published:** 2020-10-24

**Authors:** Sara B. Fournier, Jeanine N. D’Errico, Derek S. Adler, Stamatina Kollontzi, Michael J. Goedken, Laura Fabris, Edward J. Yurkow, Phoebe A. Stapleton

**Affiliations:** 1 Environmental and Occupational Health Sciences Institute, Rutgers University, 170 Frelinghuysen Rd, Piscataway, NJ 08854 USA; 2 Department of Pharmacology and Toxicology, Ernest Mario School of Pharmacy, Rutgers University, 160 Frelinghuysen Rd, Piscataway, NJ 08854 USA; 3 Molecular Imaging Center, Rutgers University, 41 Gordon Rd, Piscataway, NJ 08854 USA; 4 Department of Material Science and Engineering, School of Engineering, Rutgers University, 607 Taylor Rd, Piscataway, NJ 08854 USA; 5 Research Pathology Services, Rutgers University, Piscataway, NJ 08854 USA

**Keywords:** Nanoplastics, Translocation, Pregnancy, Maternal, Fetal, Polystyrene, Perfusion

## Abstract

**Background:**

Plastic is everywhere. It is used in food packaging, storage containers, electronics, furniture, clothing, and common single-use disposable items. Microplastic and nanoplastic particulates are formed from bulk fragmentation and disintegration of plastic pollution. Plastic particulates have recently been detected in indoor air and remote atmospheric fallout. Due to their small size, microplastic and nanoplastic particulate in the atmosphere can be inhaled and may pose a risk for human health, specifically in susceptible populations. When inhaled, nanosized particles have been shown to translocate across pulmonary cell barriers to secondary organs, including the placenta. However, the potential for maternal-to-fetal translocation of nanosized-plastic particles and the impact of nanoplastic deposition or accumulation on fetal health remain unknown. In this study we investigated whether nanopolystyrene particles can cross the placental barrier and deposit in fetal tissues after maternal pulmonary exposure.

**Results:**

Pregnant Sprague Dawley rats were exposed to 20 nm rhodamine-labeled nanopolystyrene beads (2.64 × 10^14^ particles) via intratracheal instillation on gestational day (GD) 19. Twenty-four hours later on GD 20, maternal and fetal tissues were evaluated using fluorescent optical imaging. Fetal tissues were fixed for particle visualization with hyperspectral microscopy. Using isolated placental perfusion, a known concentration of nanopolystyrene was injected into the uterine artery. Maternal and fetal effluents were collected for 180 min and assessed for polystyrene particle concentration. Twenty-four hours after maternal exposure, fetal and placental weights were significantly lower (7 and 8%, respectively) compared with controls. Nanopolystyrene particles were detected in the maternal lung, heart, and spleen. Polystyrene nanoparticles were also observed in the placenta, fetal liver, lungs, heart, kidney, and brain suggesting maternal lung-to-fetal tissue nanoparticle translocation in late stage pregnancy.

**Conclusion:**

These studies confirm that maternal pulmonary exposure to nanopolystyrene results in the translocation of plastic particles to placental and fetal tissues and renders the fetoplacental unit vulnerable to adverse effects. These data are vital to the understanding of plastic particulate toxicology and the developmental origins of health and disease.

## Background

Plastics are ubiquitous in modern society, used worldwide in a variety of applications ranging from manufacturing, packaging materials, personal products, and medical devices. Growing production and post-consumer plastic waste disposal contribute to the accumulation of plastic in landfills, waterways, and oceans [1]. In the natural environment, material fragmentation of bulk plastic waste by a combination of physical, chemical, and biological processes produces smaller particles referred to as microplastics (< 5 mm in a single dimension [2]) and nanoplastics (< 100 nm in a single dimension). Recent literature identified microplastics in atmospheric fallout [3, 4] and as a significant component of indoor air pollution [3]. These findings have raised concerns for potential adverse health effects of human nanoplastic particle inhalation [5]. In an occupational setting the potential for unintentional exposure to aerosolized micro- and nanoplastics is a critical issue. According to the United States Department of Labor Occupational Safety and Health Administration (OSHA) Regulation 1910.1000 Table Z pertaining to toxic and hazardous air contaminants, there are presently no occupational exposure limits for aerosolized micro- or nano-sized plastic particles, likely these particles are grouped into “particles not otherwise regulated” and/or “inert or nuisance dust” [6]. Current data on exposure to micro- and nanoplastics in a consumer or occupational context are very limited as the quantification of emissions in a background of ambient air particles cannot be accurately measured with existing technology [7] but may be estimated based on fragmentation of microplastics in the environment. A recent meta-analysis demonstrated that adult women are exposed to an average of 258 microplastic particles per day, of which inhalation accounts for 132 microplastic particles [8].

Unfortunately, very little is understood pertaining to the toxicology of nanoplastic particles; however, the physiological concerns of other engineered nanoparticles in a similar size range have been identified. Compared with like particles of a larger size, nanoparticles have the propensity to access deeper regions of the lung and cross biological barriers [9]. Recently, studies in mammalian models have identified maternal-to-offspring translocation of silver nanoparticles after pulmonary exposure, raising concerns for the risk of adverse health effects in dams and embryos/fetuses during pregnancy [10]. We have reported particle translocation of multi-walled carbon nanotubes (MWCNT) to the heart, kidneys, and other systemic tissues after inhalation of MWCNT aerosols in young adult male rats [11]. These studies demonstrate the probability of nanosized particles to translocate across pulmonary cell barriers to secondary organs, including the placenta. Furthermore, gold has been detected in the blood and urine of healthy human volunteers following acute inhalation of engineered gold nanoparticles [12]; also titanium was identified in the spleen and liver of young adult (12–13 weeks) and aged (19 months) rats exposed to a TiO_2_ nanostructured aerosol [13]. Although these studies identify the ionic dissociation and not the metallic particle, these outcomes may have relevance to the release of chemicals adsorbed to the surface of plastic particles in a biological environment [14].

While investigations of the impact of micro- and nanoplastic pollution in terrestrial ecosystems are limited, numerous studies have documented the effects of micro- and nanoplastics in the aquatic environment [5, 15, 16]. Mattsson et al. reported trophic transfer from prey to predator within a laboratory-simulated food chain where 53 nm polystyrene particles transferred from algae to the zooplankton *Daphnia magna,* and then to a freshwater fish [17]. Polystyrene nanoparticles (i.e., 42 nm) were identified in the yolk sac, gastrointestinal tract, liver, and pancreas of larvae and F1 embryos after maternal ingestion, providing evidence of maternal-offspring transfer in a non-placental vertebrate exposure model [18]. Cellular uptake of polystyrene nanoparticles (25 nm and 70 nm) has been reported in human alveolar epithelial A549 cells [19]. Nanopolystyrene exposure reduced cell viability, induced cell cycle S phase arrest, and up-regulation of pro-inflammatory cytokines and pro-apoptotic proteins [19]. Importantly, exposure duration, particle diameter, and concentration were key determinants of the toxicological effects of polystyrene nanoparticle exposure on alveolar epithelial cells [19].

While information about the risk of airborne micro- and nanoplastic particles to human health is limited, using an ex vivo human placental perfusion model, Wick et al. confirmed size-dependent maternal-to-fetal placental translocation of fluorescent polystyrene particles (50 nm, 80 nm, and 240 nm) [20]. Furthermore, Grafmueller et al. examined the bidirectional transfer of polystyrene particles using the ex vivo human placental perfusion model and observed placental translocation and particle accumulation in the syncytiotrophoblast of the placental tissue [21]. Investigators reported that nanoparticle translocation across the human placenta was dependent on particle physio-chemical characteristics and functionalization and was likely to involve an active, energy-dependent transport pathway [21]. While it is understood that nanoplastic particles are likely to reach the fetal tissues after maternal inhalation, the impact of maternal lung exposure to nanoplastic particles on fetal development and particle deposition within the fetus remains unclear [10, 21]. In this study, we examine the translocation and deposition/accumulation of nanopolystyrene particles in maternal and fetal tissues after a maternal pulmonary exposure in rats during late gestation. Furthermore, we assess the impact of nanopolystyrene particles on fluid flow in real-time across the live placenta using an isolated ex vivo utero-placental perfusion system.

## Materials and methods

### Polystyrene Nanobeads

Stock solutions of commercially available 20 nm rhodamine-labeled polystyrene beads (8.8 × 10^14^ particles/mL, PS20-RB-2; NanoCS, New York, NY) were suspended at a concentration of 1% in distilled water and 0.01% surfactant and sonicated for 5 min on ice prior to measurement. The size of the nanoparticles was measured with Non-Invasive Backscatter optics (NIBS) using a 4 mW, 633 nm laser. The ENM ζ-potential was also measured via Zetasizer Nano ZS. Particle size was independently verified by collaborative research partners in the Department of Materials Science and Engineering. An in-house assessment of the rhodamine-labeled polystyrene beads revealed an average particle agglomerate size of 21.86 nm ± 0.026 and a ζ-potential of − 0.0874 ± 0.195.

### Animals

Time-pregnant Sprague Dawley rats (*n* = 21) were ordered from Charles River Laboratories (Kingston, NY). Animals were delivered on gestational day (GD) 15 and allowed to acclimate within an AAALAC accredited vivarium at Rutgers University for at least 96 h. Animals were single housed in standard caging and had access to food and water ad libitum*.* Rats were randomly assigned to a treatment group upon arrival. All procedures were approved by the Institutional Animal Care and Use Committee of Rutgers University.

### Exposure

Rhodamine-labeled nanopolystyrene particles were prepared by vortexing 300 μl of manufacturer’s suspension for 2 min, followed by ultra-sonication on ice, for 5 min as previously described [22, 23]. On GD 19 rats were anesthetized with isoflurane gas (5.0% induction). Animals were placed supine on an angled board by suspending the upper incisor teeth on an incisor loop at a 45° angle. The tongue was retracted using forceps and a cotton-tipped applicator. Using a veterinary operating otoscope fitted with a speculum, the epiglottis was visualized, and a 20 gauge, 4-in. stainless steel ball-tipped oral gavage needle was inserted via the mouth to the trachea. The rats received intratracheal instillation of 300 μL (2.64 × 10^14^ particles) of nanopolystyrene suspension as described above or vehicle (0.9% NaCl). Rats were monitored after instillation and anesthesia until they regained consciousness and normal physiological activity (e.g., walking, eating, drinking, grooming, and resting).

### Fluorescent Optical Imaging

Twenty-four hours after exposure on GD 20, dams were fully anesthetized with 3–5% isoflurane in oxygen and the depilatory agent Nair™ was applied to the abdominal region to remove hair prior to imaging. The animal was transferred into the Bruker In-Vivo Multispectral (MS) FX PRO Imager (Bruker, Billerica, MA, USA) imaging chamber with nose cone attached to the manifold and placed in the prone position. The MSFX Pro Bruker detects bioluminescence, fluorescence, radio isotope, and X-ray.

A brightfield image was taken to confirm positioning and provide a snapshot/photo of the scan. The primary scans consisted of an excitation of 480 nm with an emission of 535 nm for a 1-min exposure. Later scans consisted of an excitation of 550 nm with an emission of 600 nm. For these scans, the detectable light refracting off the contrast was recorded. The final scan in this series was an X-ray of the sample that assisted with co-registration of the signal with organ tissues. Following live imaging, animals were sacrificed by removal of the heart according to the Rutgers IACUC approval. Maternal tissues, periparturient fetuses, and fetal tissues were harvested and placed on a polycarbonate tray. After tissue scans were complete, the regions of interest were measured using Bruker MSFX PRO Image software.

Hyperspectral-enhanced darkfield microscopy: Formalin fixed fetal tissues were processed, embedded in paraffin, and sectioned to 4 μm. Slides were visualized via transmitted darkfield hyperspectral images and data captured using CytoViva optics at 60x magnification with oil objective. Dual Mode Fluorescence (DMF) and full fluorescence images were captured with Texas Red excitation filter and triple pass emission filter for further particle confirmation. Data was processed using ENVI 4.8 (CytoViva, Inc., Auburn, AL).

### Placental Isolation and Perfusion

A separate cohort of naïve gravid rats (*n* = 14) were anesthetized with isoflurane (5% induction and 3% maintenance) on GD 20. The right uterine horn was isolated, removed, and placed into a dish of cold (4 °C) physiological salt solution (PSS). Briefly, the uterine horn was dissected, placental unit was identified, amniotic sac opened, fetus removed, and umbilical vessels were ligated and unraveled as previously described [24, 25]. The placental unit was removed and placed into a modified isolated vessel chamber (Living Systems Instrumentation, Burlington, VT) filled with warmed (37 °C), oxygenated (21% O_2_–5% CO_2_–74% N_2_), circulating PSS. The placental vasculature (uterine artery and umbilical artery and vein) were secured to glass pipettes or 26 gauge, 4-in. stainless steel blunt needles, respectively. The uterine artery was perfused with a peristaltic pump at 80 mmHg and the umbilical artery was perfused at 50 mmHg. Rhodamine-labeled nanopolystyrene particles were prepared as described above by vortexing 1 mL of manufacturer’s suspension for 2 min, followed by ultra-sonication on ice, for 5 min as previously described [22, 23]. After a 30-min equilibration and 10-min baseline, a bolus of 900 μL (7.92 × 10^14^ particles/mL) of nanopolystyrene particles were slowly injected into the uterine artery. Effluents were collected and weighed from the distal uterine artery and umbilical vein cannula at 10-min intervals for a total of 180 min. The remaining fluid within the stainless-steel needle cannulating the umbilical vein was collected.

### Quantification of Nanopolystyrene particles

Twenty five μL of effluent from each sampling time point was pipetted in duplicate on a 96-well clear bottom plate. The positive control was identified as 25 μL of stock solution of 20 nm rhodamine-labeled polystyrene beads and the negative control as PSS only. All samples were diluted by adding 100 μL of PSS into each well. Fluorescence was measured by a spectrophotometer at 546/575 nm (excitation/emission) using a SpectraMax M3 fluorescent microplate reader (Molecular Devices, Sunnyvale, CA). Data were collected using SoftMax Pro 6.3 software.

To confirm that the rhodamine tag remained attached to the polystyrene beads throughout the perfusion, maternal and fetal effluents were pooled together for 4 representative experiments. The samples were centrifuged at 100,000 x g for 1 h in an ultracentrifuge (Beckman Coulter Max-XP tabletop Ultracentrifuge) to pellet polystyrene ENM. Twenty five μL of supernatant was removed from each sample and placed in a 96-well clear bottom plate and read at 546/575 nm (excitation/emission) using a SpectraMax M3 fluorescent microplate reader (Molecular Devices, Sunnyvale, CA). Data were collected using SoftMax Pro 6.3 software.

### Histology

Representative placentas from the perfusion experiments were fixed in 10% neutral buffered formalin, processed and sectioned to 4 μm. Hematoxylin and eosin (H&E) stained slides were assessed by an ACVP board-certified veterinary pathologist.

### Statistics

Outliers were identified using Grubb’s test and removed from the data presented in Table 1. All other results are presented in their entirety. All data were analyzed by Student’s t-test using MS Excel. Statistical significance was set to *p* < 0.05 and is indicated with an asterisk (*). Trends were identified as *p* < 0.10 and are indicated with a (T). ANCOVA analyses were also run using Sigma Plot 13.0 to assess if pup and placental weights were impacted by litter size.

**Table 1:** Effect of maternal nanopolystyrene pulmonary exposure on litter characteristics

Treatment	***n***	Maternal Weight (g)	Number of Fetuses per Litter	Fetal Weight (g)	Placental Weight (g)	Placental Efficiency	Number of Resorption Sites
**Saline**	14	358 ± 12	13.1 ± 0.4	2.71 ± 0.05	0.48 ± 0.01	5.57 ± 0.26	0.42 ± 0.14
**PS**	11	382 ± 21	12.6 ± 0.6	*2.51 ± 0.06	*0.43 ± 0.01	5.79 ± 0.22	*1.13 ± 0.30

Values are shown as mean ± SEM

*n* number of dams. Statistics were analyzed with a one-way analysis of variance (*p ≤ 0.05)

## Results

### Exposure Dosimetry: extrapolation to real-world conditions

**Fig. 1:**
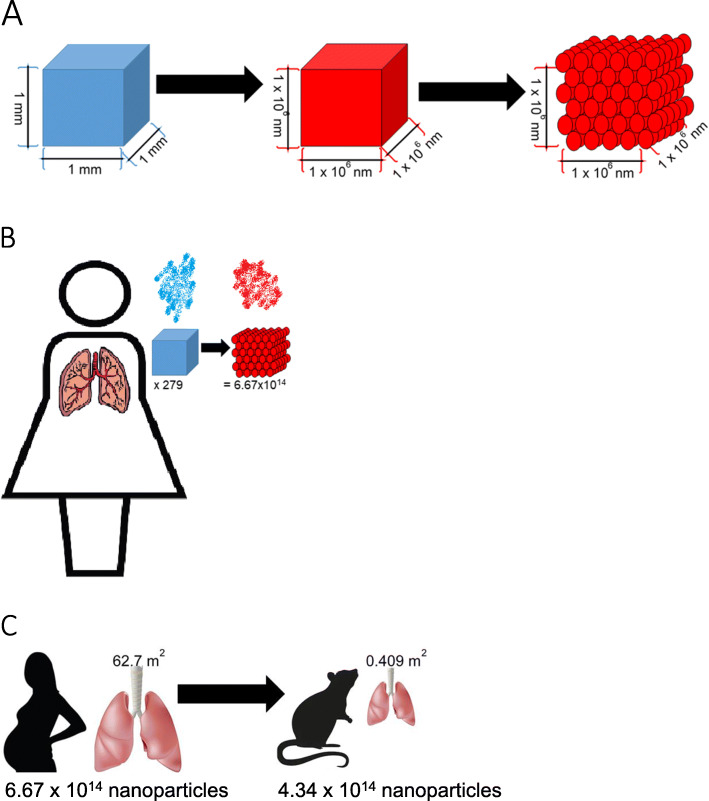
Schematic of nanoplastic exposure and dosimetry. **a** We utilized a 1 mm^2^ microparticle as a representative microplastic (blue). The extrapolation of this microplastic microparticle to a nanoparticle is 1 × 10^6^. Our representative nanopolystyrene nanobeads are spherical and 21 nm in diameter (red). Therefore, there would be 2.39 × 10^14^ nanopolystyrene beads in a single plastic microparticle. **b** Cox et al. identified that women inhale an average of 132 microplastics. The upper bound of this measurement (279 microplastics), is more representative of exposure for pregnant women. The calculated dosage is 6.67 × 10^16^ nanopolystyrene beads. **c** The surface area of the Sprague Dawley rat lung is significantly smaller (0.409 m^2^) compared with the human lung (62.7 m^2^). The calculated dose for a laboratory rat is 4.34 × 10^14^. The exposure dose used in these studies was 2.64 × 10^14^ nanopolystyrene beads

The concentration of microplastic particles has been measured in both atmospheric and indoor air [3, 4, 8]. Unfortunately, nanosized particles have not been directly measured in real-world conditions; because of their small size, they generally escape traditional containment and filtering systems. Therefore, to extrapolate nanopolystyrene particle dosage, we considered an 1 mm^3^ atmospheric microparticle and mathematically converted this to nanosized particles representative of our spherical 20 nm polystyrene beads. Consequently, a single microparticle represents 2.39 × 10^14^ nanopolystyrene particles (Fig. 1a). Cox et al. reported that the average women inhales 132 microplastic particles per day [8]. Given that maternal minute ventilation, or the volume of gas inhaled, increases by up to 48% during pregnancy while the respiration rate remains unchanged [26], it is likely that daily total exposure is closer to the upper bound of 279 microplastics identified in the study [8]. These data suggest that the average pregnant woman could be exposed to 6.67 × 10^16^ nanoplastic particles per day (Fig. 1b). When the surface area of the lung between human (62.7 m^2^) and our laboratory rat (0.409 m^2^) model [27] is considered, the appropriate experimental exposure amounts to 4.34 × 10^14^ nanoplastic particles (Fig. 1c). This value is much greater than the exposure dose of 2.64 × 10^14^ nanoplastic particles and therefore, the exposure dose used in this study is within real-world considerations.

### Fluorescent optical imaging

**Fig. 2:**
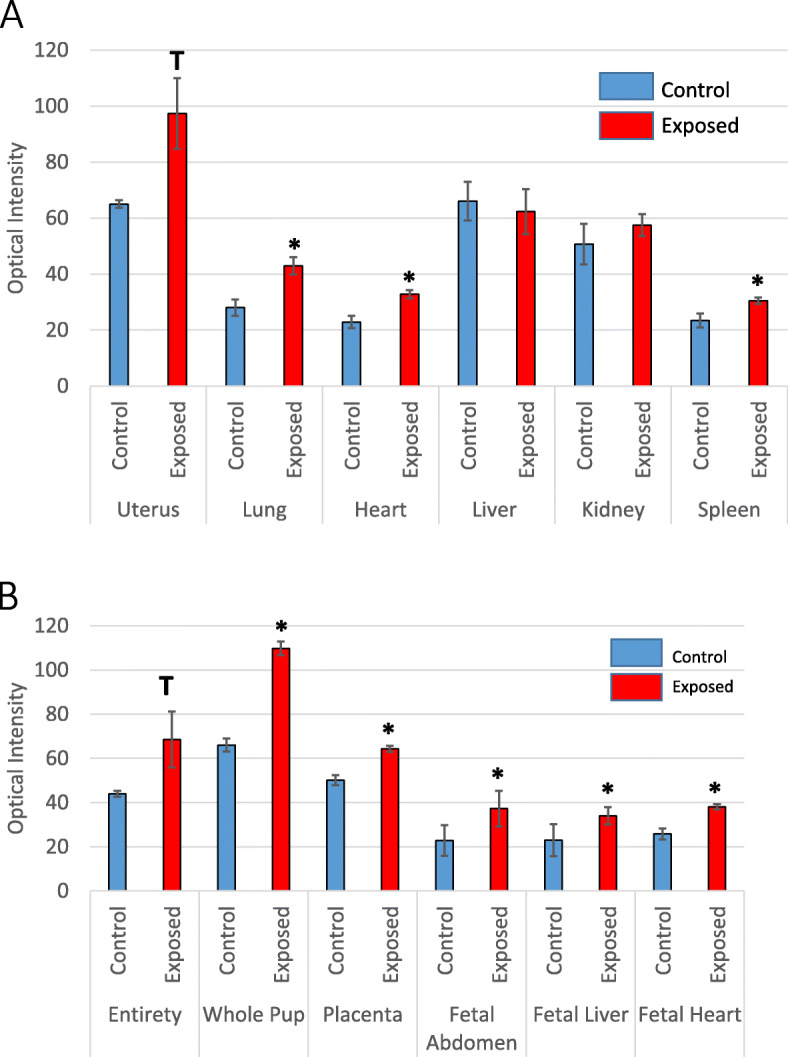
Optical images of maternal and fetal tissues. Graphical representation of the optical intensities between **a** maternal and **b** fetal control and exposed tissues. *n* = 6–8 pregnant rats. Values are shown as mean ± SEM. Statistics were analyzed with a Student’s t-test. (*p ≤ 0.05; T ≤ 0.10)

Primary whole animal scan yielded null results as the skin was too dense to visualize any fluorescence (data not shown). Graphical representations of optical intensities are represented for maternal tissues in Fig. 2a and fetal tissues in Fig. 2b. These data indicate significant nanopolystyrene deposition in the maternal lung, heart, spleen, compared to controls and a trend toward significance in the gravid uterus. Deposition of polystyrene was elevated in all exposed fetal tissues that were evaluated. Images obtained from secondary scans of fetal tissues revealed significantly elevated levels of polystyrene in GD 20 fetuses, fetal abdomens, and isolated livers compared with controls. There was an elevated trend in the fetus and placenta in its entirety within the amniotic sac, isolated placenta, and isolated fetal hearts. These studies indicate nanopolystyrene particle translocation from the maternal lungs to systemic tissues, including the fetus and fetal organs.

### Hyperspectral Darkfield microscopy

**Fig. 3:**
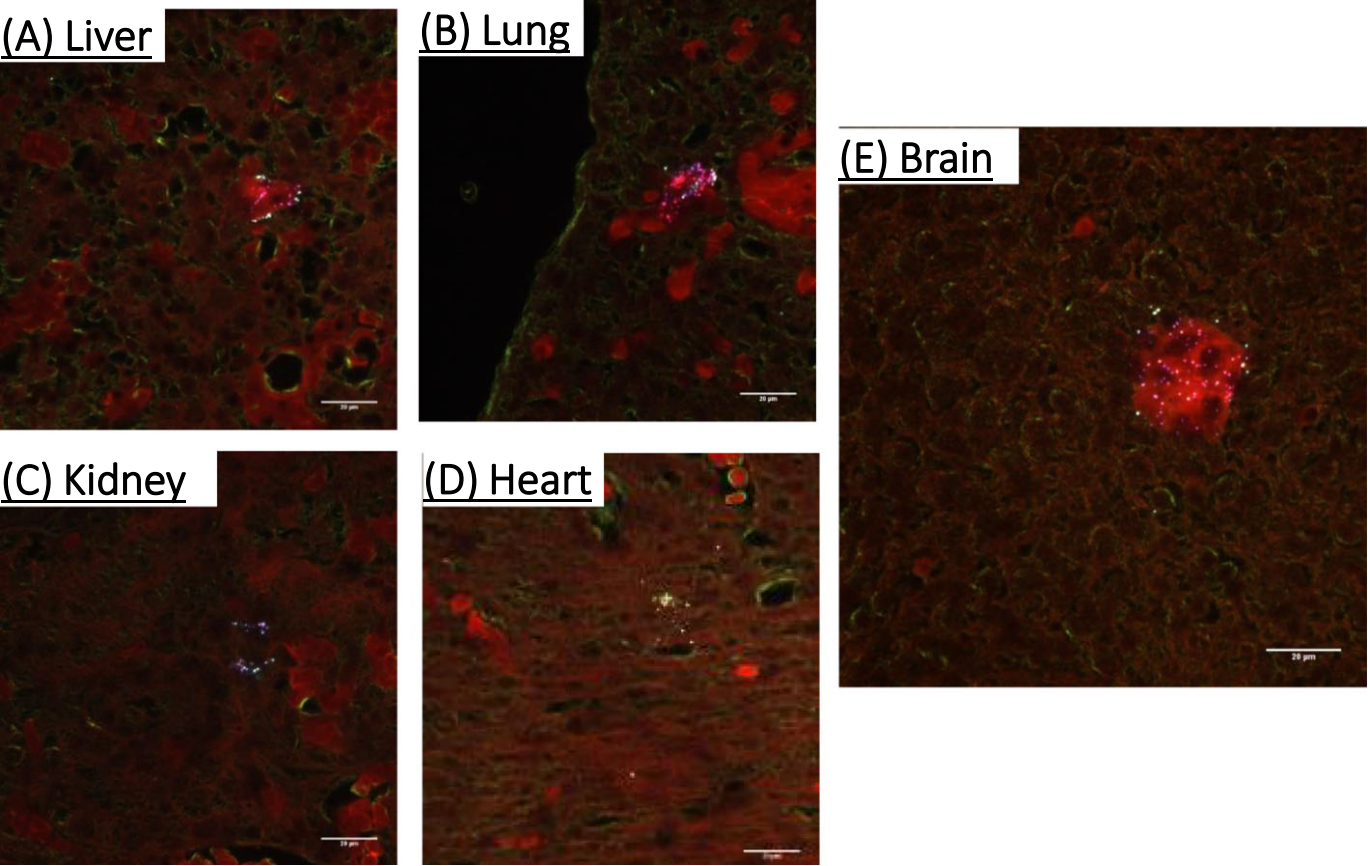
Identification and visualization of nanopolystyrene particle deposition within the fetal tissues placenta after material pulmonary exposure using enhanced hyperspectral microscopy (CytoViva, Inc.). These tissues include fetal **a** liver, **b** lung, **c** kidney, **d** heart, and **e** brain. *n* = 3 fetuses from 3 different pregnant rats. Polystyrene nanoparticles are identified as white specs within the images

Enhanced darkfield imaging of fetal tissue sections readily demonstrated polystyrene nanoparticle deposition in Fig. 3. Polystyrene nanoparticles were visualized in the fetal liver, lung, kidney, heart, and brain. In representative images, these particles appear as white dots/spots. These studies further demonstrate nanoplastic particle deposition within fetal tissues.

### Placental perfusion

**Fig. 4:**
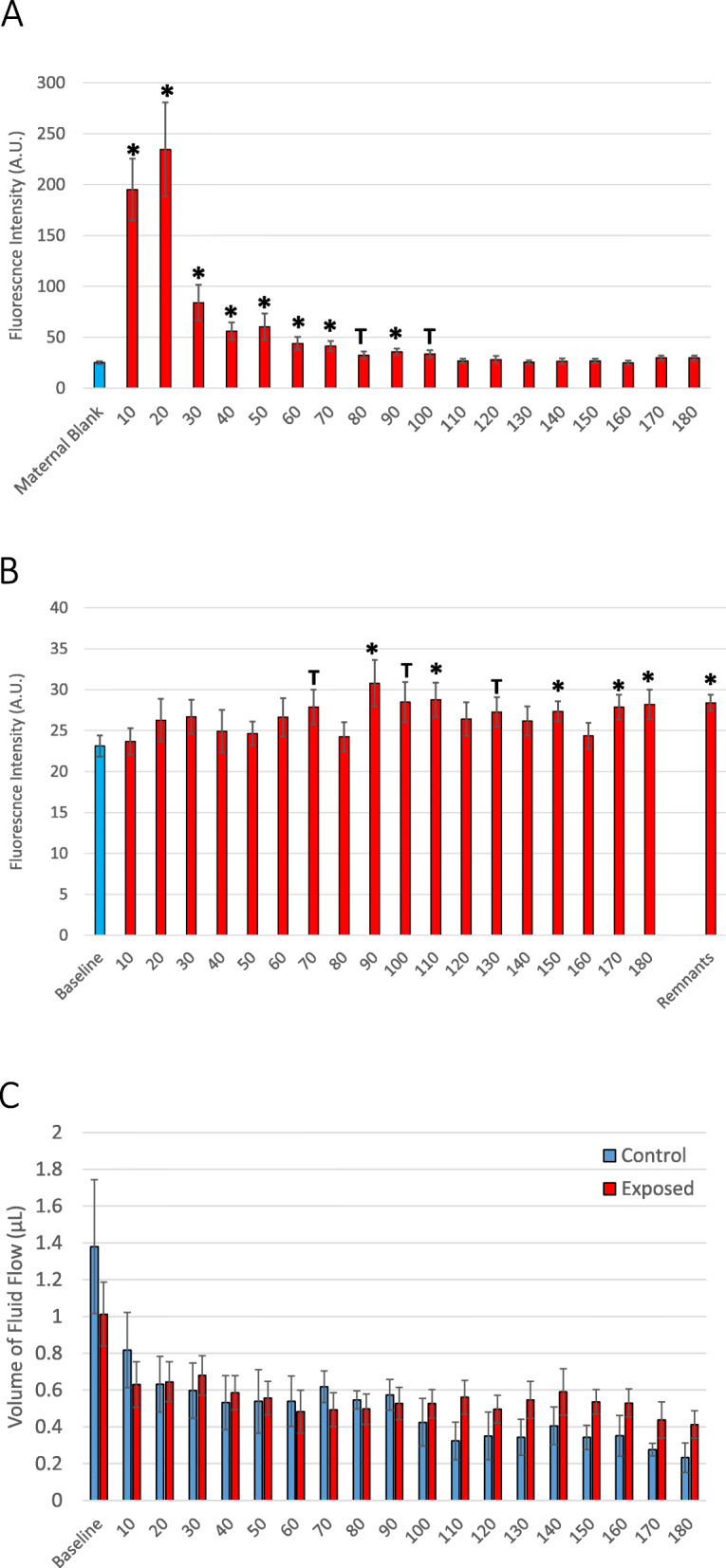
Identification of rhodamine-labeled nanopolystyrene bead translocation based on increased fluorescence through the **a** distal uterine effluent and **b** umbilical vein effluent over time. *n* = 9–24. **c** Time-course of fluid flow through the umbilical vein between saline (black) and nanopolystyrene (red) infused placenta. *n* = 6–8. Values are shown as mean ± SEM and presented as percent above baseline. Statistics were analyzed with Student’s t-test (*p ≤ 0.05; T ≤ 0.10)

Polystyrene nanoparticle transfer through the maternal vasculature from the proximal to the distal uterine artery was confirmed within 10 min of bolus infusion (Fig. 4a). Nanoparticle transfer across the maternal uterine artery peaked at 20 min, remained significantly above baseline for 70 min, and elevated for 100 min. Effluent fluorescence returned to baseline levels after 100 min. Moreover, elevated concentrations of polystyrene nanoparticles were detected in umbilical effluent within 90 min of uterine artery bolus infusion (Fig. 4b). Concentration of the nanoparticles in the umbilical effluent were significantly high after 150 min through 180 min after infusion and significant concentrations remained in the umbilical cannula after 180 min of perfusion. There were no significant differences in fluid flow through the umbilical vein between saline perfused placenta (control) and those exposed to nanopolystyrene after the 180-min perfusion (Fig. 4c). Placentae were evaluated after perfusion where no histopathological alterations were identified.

### Litter characteristics

Maternal and fetal parameters including maternal weight, litter size, fetal weight, placental weight, placental efficiency, and total number of sites of resorption are reported in Table 1. In treated dams, fetal and placenta weights were significantly lower in the exposed group compared with control. ANCOVA analyses confirmed that these results were not the product of litter size variation. Number of resorption sites in the exposed group was also significantly greater compared with control. There were fewer fetuses in dams treated with nanopolystyrene particles, however, this difference did not reach significance in our cohort.

## Discussion

In this study we identified the translocation of nanopolystyrene particles from the maternal lungs, across the placenta, into fetal tissues. Elevated fluorescence from our rhodamine-labeled particles was measured in the maternal lung, heart, spleen, and uterus and fetal placenta, liver, and heart. Nanopolystyrene particles were observed in the fetal liver, lung, kidney, heart, and brain in late-stage pregnancy using dark-field microscopy. Furthermore, using an ex vivo placental perfusion system, we observed the transfer of nanopolystyrene particles from the maternal uterine circulation, across the placenta to the fetal circulation. As is pertains to fetal health, we observed reduced fetal weight, reduced placental weight, and an increase in reabsorption sites 24 h after maternal nanopolystyrene particle pulmonary exposure.

In this study, we visualized nanopolystyrene translocation from the maternal lungs to the fetal compartment and deposition in the fetal, liver, heart, kidney, and brain on GD 20, within 24 h of maternal exposure. Our study represents a snapshot of time during gestation, providing evidence that nanoplastic particles can reach fetal tissues after maternal pulmonary exposure. As it pertains to nanoplastic particle deposition, it remains unclear if the nanopolystyrene particles have been taken up by the fetal cells, remain in the fetal vasculature, migrate to the interstitial space, or are returned to the maternal circulation. It is plausible that these particles would remain in the fetus after birth as the nanoplastic particles passaged across the placental barrier and may be taken up by fetal cells. Endothelial cell exposure to engineered nanomaterials enhances endothelial barrier permeability [28–30], which offers accessibility to the interstitial space between cells within systemic tissues. Reports pertaining to the development and function of the blood brain barrier in a fetus are inconclusive [31, 32]. Therefore, the blood-brain barrier may not yet be fully formed, rendering the fetal brain susceptible to particle sedimentation. We, and others, have identified that maternal exposures to metallic and carbonaceous ENM during gestation can initiate developmental onset of disease within the maturing fetus. In laboratory studies, young and adult offspring have been reported to exhibit coronary dysfunction [33–38], vascular perturbations [38, 39], negative reproductive health outcomes [40–42], and neurological outcomes [43–45] after maternal inhalation of engineered nanomaterials during pregnancy. It is also plausible that these findings represent a snapshot of time, wherein the particles reach the fetal tissues within 24 h of exposure but are removed from the fetal circulation prior to birth. Therefore, particle deposition during fetal development may impact offspring health after birth and into adulthood.

Furthermore, the uptake and passage of nanosized materials is highly dependent on the physio-chemical properties of the particles including size, functionalization, chemical construct, and surface charge [5]. Cellular uptake and subsequent toxicity of nanoplastic particles is dependent on the unique protein and chemical corona that forms on the surface during contact with biological fluids (e.g. pulmonary surfactant, interstitial fluid, plasma) and environmental chemicals; in the case of plastics these chemicals may be adsorbed and serve and a vehicle for chemical transport [46]. Chemical additives adsorbed to the surface or added to plastics during the polymerization process can leach or be transferred from polystyrene products with normal use. These additives may include carcinogens or endocrine disrupting factors (e.g., vinyl chloride, phthalates) [47, 48]. Fundamental studies of plastics toxicology identify and refer to the potential for chemical leakage from polystyrene products after the addition of hot, cold, or boiling water [49]. Recently, studies identify the propensity for polycyclic aromatic hydrocarbons, specifically pyrene, to dissociate from after aquatic exposure to microplastic particles in a biological environment [14]. Together, these outcomes indicate the possibility of chemical release from particles within an organism. Fetal nanoplastic deposition could lead to life-long localized low-level exposure to these additives or adsorbed chemicals. Future studies are planned to examine chemical release from plastic nanomaterials within a biological environment and to assess the impact of a chronic exposure to nanoplastic particles on fetal growth and development are also required for a comprehensive understanding of the health hazards associated with airborne nanoplastics.

We quantified an elevation in fluorescently labeled nanopolystyrene particles in the umbilical vein within 90 min of bolus infusion to the uterine artery. These levels were significantly higher within 150 min of exposure. These results confirm the capacity of 20 nm nanopolystyrene plastic particles to pass from the maternal to the fetal compartment. Interestingly, while fluid flow from the maternal to fetal compartment decreased after both saline control and nanopolystyrene injection, this was not to significance. This suggests no reduction in blood flow through the placenta within 180-min of particles reaching the uterine artery.

Similarly, Grafmueller et al. demonstrated the placental transfer of fluorescently-labeled nanopolystyrene particles from the maternal to the fetal compartment [20, 21]. Upon further study utilizing the ex vivo human placental perfusion model, the authors identified a bidirectional, size-dependent transfer of nanopolystyrene beads without cytotoxicity [21]. In this study, heightened polystyrene particle transfer from the fetal to the maternal compartment was observed instead of a concentration equilibrium evident of passive transport [21]. Therefore, Grafmueller et al. speculated that nanopolystyrene translocation across the placenta likely involves energy-dependent uptake, material transfer, and particle efflux as opposed to passive transport [21]. It is recognized that the concentration of particles that translocate from the primary site of exposure to the fetal compartment and tissues is low [50] and that the human placental perfusion methodology is not without limitation [51].

The results of our current study also corroborate data from previous nanotoxicological investigations from our group and others that have shown reduced fetal weight, or restricted fetal growth, after chronic maternal inhalation of nano-titanium dioxide particles [39, 52]. We have previously postulated that diminished fetal development after maternal nanoparticle exposure in our studies may be associated with indirect vascular deficiencies leading to uteroplacental ischemia in late-stages of pregnancy [39, 53, 54]. These vascular deficiencies are also evident after a single pulmonary exposure to nano-titanium dioxide particles during pregnancy [53, 54]. We have also observed decreased fluid flow during ex vivo placental perfusion after gold nanoparticle infusion [24]. Interestingly, we identified reductions in fetal weight, placental weight, and increased number of fetal reabsorptions after an acute maternal nanoplastic pulmonary exposure in this study, but did not observe a reduction in fluid flow to the fetal compartment after direct infusion of nanopolystyrene particles into the uterine artery. Unfortunately, despite more recent studies, these limited data do not provide enough evidence to make definitive statements regarding adverse gestational or litter effects after maternal pulmonary nanomaterial exposure [50]. Therefore, future studies of uteroplacental function are necessary to clarify if the discrepancies in these results are based on differences between single, acute, or chronic nanopolystyrene exposures or if these changes are mediated only by metallic nanomaterials.

## Conclusion

Collectively, these results identified the impact of a pulmonary exposure to an environmentally relevant dose of nanopolystyrene and examined maternal and fetal parameters and the translocation of plastic particles to, and deposition within fetal tissues. These data are vital to the understanding of plastic particulate toxicology and the developmental onset of health and disease. Future studies are required to provide a more detailed exploration of organ-specific toxicity and the implications of nanoplastic exposure on reproductive potential and fetal development.

## Data Availability

All data generated or analyzed during this study are included in this published article.
